# Development and validation of microsatellite markers for *Brachiaria ruziziensis* obtained by partial genome assembly of Illumina single-end reads

**DOI:** 10.1186/1471-2164-14-17

**Published:** 2013-01-16

**Authors:** Pedro IT Silva, Alexandre M Martins, Ediene G Gouvea, Marco Pessoa-Filho, Márcio E Ferreira

**Affiliations:** 1 Embrapa Recursos Genéticos e Biotecnologia, Genetics Lab, PO Box 02372, Brasília, CEP 70770-917, Distrito Federal, Brazil; 2 Departamento de Biologia Celular, IB - Universidade de Brasília (UnB) Campus Universitário Darcy Ribeiro, Asa Norte, Brasília, CEP 70910-900, Distrito Federal, Brazil; 3 Embrapa Cerrados, PO Box 08223, Brasília, CEP 73310-970, Distrito Federal, Brazil; 4 Current address: Dupont Pioneer, Palmas, Tocantins, Brazil

## Abstract

**Background:**

*Brachiaria ruziziensis* is one of the most important forage species planted in the tropics. The application of genomic tools to aid the selection of superior genotypes can provide support to *B. ruziziensis* breeding programs. However, there is a complete lack of information about the *B. ruziziensis* genome. Also, the availability of genomic tools, such as molecular markers, to support *B. ruziziensis* breeding programs is rather limited. Recently, next-generation sequencing technologies have been applied to generate sequence data for the identification of microsatellite regions and primer design. In this study, we present a first validated set of SSR markers for *Brachiaria ruziziensis*, selected from a *de novo* partial genome assembly of single-end Illumina reads.

**Results:**

A total of 85,567 perfect microsatellite loci were detected in contigs with a minimum 10X coverage. We selected a set of 500 microsatellite loci identified in contigs with minimum 100X coverage for primer design and synthesis, and tested a subset of 269 primer pairs, 198 of which were polymorphic on 11 representative *B. ruziziensis* accessions. Descriptive statistics for these primer pairs are presented, as well as estimates of marker transferability to other relevant brachiaria species. Finally, a set of 11 multiplex panels containing the 30 most informative markers was validated and proposed for *B. ruziziensis* genetic analysis.

**Conclusions:**

We show that the detection and development of microsatellite markers from genome assembled Illumina single-end DNA sequences is highly efficient. The developed markers are readily suitable for genetic analysis and marker assisted selection of *Brachiaria ruziziensis*. The use of this approach for microsatellite marker development is promising for species with limited genomic information, whose breeding programs would benefit from the use of genomic tools. To our knowledge, this is the first set of microsatellite markers developed for this important species.

## Background

The area planted with forage crops in the tropics extends for hundreds of millions of hectares. In Brazil alone, the forage cropped land exceeds 100 M ha [1], where four brachiaria species (*B. brizantha, B. decumbens, B. ruziziensis* and *B. humidicola*) cover 85% of the cultivated pastures [2]. Only a few apomictic brachiaria clones occupy tens of millions of hectares in the country [3], what represents a high risk of genetic vulnerability for forage production. This risk could be reduced with the increased use of genetic diversity conserved in germplasm banks in order to generate recombinant genotypes in breeding programs. The development and adoption of new brachiaria cultivars with a broad genetic base is crucial for the diversification of forage pasture in the tropics. The development of new cultivars must be a dynamic process, providing the pasture production sector with increasing genetic diversity.

Among the four brachiaria species most cultivated in Brazil, ruzigrass (*Brachiaria ruziziensis*, 2n=2x=18) stands out as a diploid species with sexual reproduction. Polyploid brachiaria species such as *B. brizantha, B. decumbens* and *B. humidicola* typically present apomictic reproduction, a disadvantage for breeding programs that rely on sexual crosses and recombination for superior genotype selection. Ruzigrass has good forage quality, fast growth in the beginning of the rainy season and is readily adaptable to forest-crop-livestock integration systems, not only for animal feeding (green pasture or hay) but also as soil coverage for no-till farming. After tetraploidization, ruzigrass plants can be crossed with other brachiaria species, making the inter-specific introgression of genes possible. Seed production is uniform, since flowering occurs only once a year. This favors a decrease in seed production costs and an increase in seed quality. The elimination of the seed shattering trait is an essential move in enabling full domestication of *B. ruziziensis*, and will contribute to production of high quality seeds, turning *B. ruziziensis* into an essentially agricultural crop.

Ruzigrass has a relatively small genome (~600 Mbp [4]), similar to other model cereal species, such as rice (430 Mbp) and sorghum (700 Mbp). This enables genome analysis initiatives and the development of molecular tools to support breeding programs. In contrast, tetraploid brachiaria species (e.g. *B. decumbens, B. brizantha*) have larger and more complex genomes (> 1,600 Mbp). Therefore, ruzigrass has great potential to be used in breeding programs for pasture diversification, especially in combination with genomic tools aiding the selection of superior genotypes.

The employment of these genomic tools would favor a more dynamic development of new cultivars for this species. However, there is a lack of information about the *B. ruziziensis* genome. Little or nothing is known about the number of genes, distribution of gene families, abundance and diversity of retro-elements, QTL localization of traits of economic importance, genome collinearity with model species, or abundance of repetitive sequences. Genomic tools, such as molecular markers (e.g. microsatellites and SNPs), to support breeding programs are simply not available.

Traditional methods for the identification of microsatellite markers usually demand the construction of small-insert genomic libraries, colony selection by microsatellite-containing probe hybridization, sequencing of selected clones, primer design for suitable flanking regions, and assessments on the marker polymorphism by PCR analysis on a germplam sample. Later on, methods employing microsatellite-enriched genomic libraries diminished costs, time and workload necessary for marker development [5-7].

More recently, research groups have been applying next-generation sequencing technologies to generate sequence data for the genome identification of microsatellite regions and primer design [8-12]. For this purpose, both genomic DNA and genic regions (using cDNA libraries) have been used as templates for sequencing. The impact of this approach on microsatellite maker development is evident: partial genomic surveys using even fractions of a lane on next-generation sequencing machines allow the discovery of thousands of potentially amplifiable microsatellite regions which can be selected for primer design [13]. This is a promising approach for species with limited genomic information, whose breeding programs would greatly benefit from the use of genomic tools.

In brachiaria, marker development initiatives so far used microsatellite enriched libraries to obtain SSRs for the species *B. brizantha*[14-16] and *B. humidicola*[14,17,18]. In summary, around 28 markers were polymorphic in *B. brizantha*, and 65 in *B. humidicola*. These authors tested the transferability of these markers to other brachiaria species, and the rates of successful amplifications varied with the target species. At least 12 out of the 28 markers developed from *B. brizantha* produced amplified PCR products in *B. ruziziensis* DNA*.* Similarly, PCR products were observed on 13 out of 65 microsatellites developed from *B. humidicola*, when these were tested on *B. ruziziensis* DNA. No information on descriptive statistics such as polymorphic information content (PIC), allelic variation or heterozygosity estimates has been provided for these markers when tested on ruzigrass accessions.

In this study, we present a first set of 500 SSR markers developed for *Brachiaria ruziziensis*, selected from a *de novo* partial genome assembly of single-end Illumina reads. Descriptive statistics for 198 of these markers are provided. A set of 11 multiplex panels for the simultaneous amplification of the 30 most informative markers (ranked by their Polymorphism Information Content) is made available. These markers will be readily useful for the *B. ruziziensis* breeding program, aiding in areas such as germplasm characterization, construction of linkage and QTL maps, gene flow and mating system evaluation, and marker assisted selection.

## Results

### Number of SSR loci initially detected in the ruzigrass genome

We restricted our search for microsatellite-containing regions to perfect di- tri- and tetranucleotide motifs only. After partial *de novo* genome assembly, a total of 139,098 perfect microsatellite loci were detected (Table 1). In order to select loci for subsequent primer design, we looked for perfect microsatellites in contigs >200 pb with a minimum 10X coverage. This reduced the number of regions to 85,567.

**Table 1:** **Summary of Illumina single**-**end read sequence data and *de novo *assembly**; **perfect di**-, **tri**- **and tetra**-**nucleotide SSR loci for *Brachiaria ruziziensis***

	**All contigs**	**Only contigs** >**200 bp**
Reads #	186,764,108	186,764,108
Read average length bp	76	76
Reads bp	14,194,072,208	14,194,072,208
Mapping Parameters (LF - SIM)	0.5 - 0.8	1.0 - 1.0
Reads Matched	179,690,233	68,644,823
Matched bp	13,656,457,708	5,217,006,548
Contigs #	1,113,797	419,751
N50	585	954
Contigs bp	367,553,010	277,588,081
Average coverage	37x	18,8x
Contig average length	330	661
Perfect microsatellite sequences	139,098	85,567
Di-nucleotides	13,127	3,919
Tri-nucleotides	113,098	72,902
Tetra-nucleotides	12,892	8,746

### Most frequent motif types and repeat numbers

**Figure 1:**
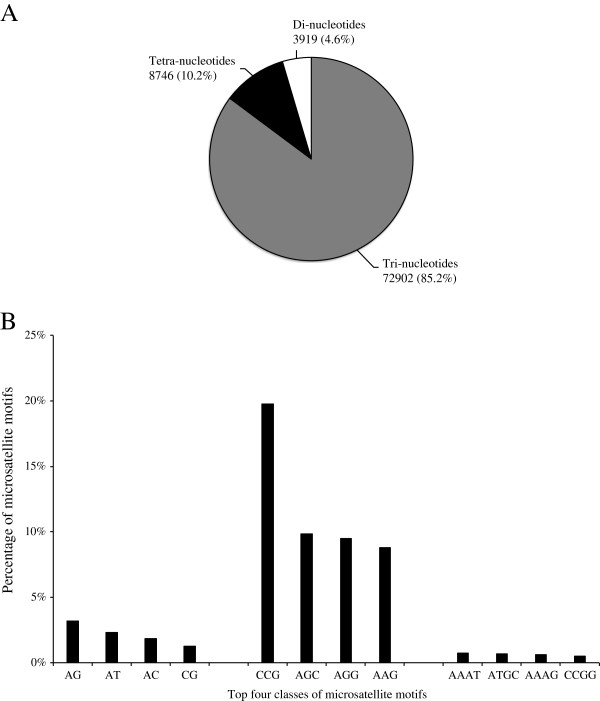
**(A)Distribution of di-**, **tri-**, **and tetra**-**nucleotide microsatellites on contigs with a minimum 10X coverage;(B)Distribution of most frequent repeat motifs on contigs with a minimum 10X coverage.**

Tri-nucleotide repeats were the most abundant class of microsatellites (72,902 regions) detected in the partially assembled ruzigrass genome, followed by tetra-nucleotide (8,746) and di-nucleotide repeats (3,919) (Figure 1A). AG, CCG, and AAAT were the most frequent types of microsatellite sequences detected on each class (Figure 1B). The most frequent tri-nucleotide repeat motif (CCG) was particularly the most abundant one, comprising 19.8% of the perfect microsatellite regions detected on contigs with at least 10X coverage. Di- and tetra-nucleotide repeat motifs, on the other hand, had a more balanced distribution among different classes. The average number of repeats was three for tri- and tetra-nucleotides, and six for di-nucleotides.

### Synthesized primer pairs

**Figure 2:**
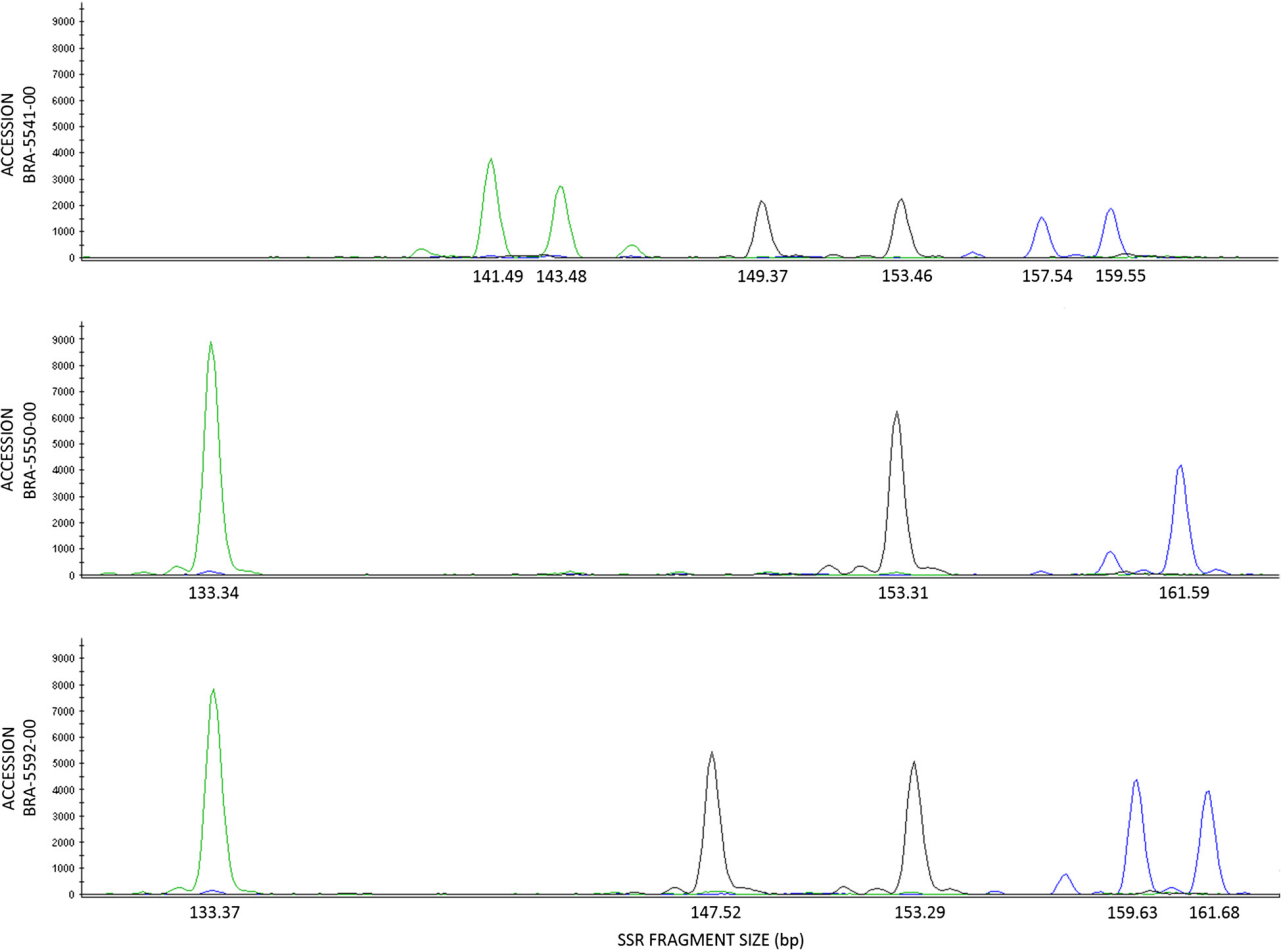
**Electropherograms of a mutiplex panel showing amplification patterns of three Brz markers(Brz0059**, **green**; **Brz0069**, **black**; **Brz0047**, **blue),in three ruzigrass accessions(BRA**-**5541**-**00**, **BRA**-**5550**-**00**, **and BRA**-**5592**-**00).**

A total of 1,135 perfect microsatellite loci were detected in contigs with a minimum 100X coverage. We selected 500 loci at random for primer design and synthesis, which were given the “Brz” prefix. Additional file 1: Table S1 includes information regarding their forward and reverse primer sequences, their melting temperatures, repeat motifs, and expected product sizes. A subset of these loci was labeled with fluorescent dyes and multiplexed in order to test their efficiency on genotyping ruzigrass accessions. We tested 92 multiplex panels containing 269 primer pairs (panels contained up to three loci). Successful genotyping of 239 of these loci was achieved, while the remaining 30 loci presented either difficult interpretation of genotyping data, or absence of amplified products. However, no PCR optimization attempts were made for these loci. This represents a minimum 88.9% success rate of PCR amplification in unoptimized conditions for microsatellite loci generated from this partial *de novo* genome assembly. Among those 239 markers presenting coherent, interpretable amplified products, 198 (82.8%) markers were polymorphic when tested on 11 diverse African-derived ruzigrass accessions. If we consider the loss of microsatellite markers in the whole process, at least 73.60% of the 269 tested loci represent polymorphic, informative markers which can be readily applied to ruzigrass germplasm characterization and breeding. Figure 2 shows an example of electropherogram for one of the tested panels on three ruzigrass accessions.

### Descriptive statistics for each SSR marker

Genotyping of 11 ruzigrass accessions with these 198 markers detected 835 alleles. The initial database of allele frequencies in *Brachiaria ruziziensis* shows 8.38% of rare alleles (with a frequency < 0.05), 64.07% of intermediate alleles (0.05 < frequency < 0.30) and 27.54% of abundant alleles (frequency > 0.30). Additional file 2: Table S2 presents the descriptive statistics information regarding these polymorphic markers.

The number of observed alleles for all polymorphic SSR markers ranged from 2 to 12, with an average value of 4.22 alleles per locus. Their expected heterozygosity (He) values ranged from 0.09 to 0.84, with an average of 0.518. Observed heterozygosity (Ho) values ranged from 0 to 1, with an average of 0.410. The Polymorphism Information Content (PIC) values ranged from 0.09 to 0.87, with an average of 0.519.

Expected product sizes for each microsatellite marker are based on sequence information generated by the *de novo* assembly process. We checked whether the size ranges for the polymorphic loci included their expected product size. This was true for 70.2% of the loci (139 out of 198). The proportion of markers that generated amplicons within 10% of their expected sizes was 95.9% (190 out of 198). No markers presented amplicons 90% larger or smaller than expected.

We ranked the 30 most informative markers regarding their PIC values and assembled them into 11 multiplex panels for fast ruzigrass genotyping. The average PIC value for the 30 markers was 0.803, varying from 0.74 to 0.87. Table 2 shows these panels and markers and their respective primer sequences and descriptive statististics.

**Table 2:** A set of 11 multiplex panels including the 30 most informative ruzigrass microsatellite markers

**Panel**	**Marker**	**Dye**	**Forward Primer**	**Reverse Primer**	**Allele No**	**Observed size ranges**	**He**	**Ho**	**PIC**
1	Brz0182	NED	ACGTTATTGGACTTGGGTGA	AGCCTGACCAAATTCTTGTG	10	252-328	0.823	0.545	0.868
	Brz0097	HEX	TAATTTGTTCCACCCACAGG	GTGACAGAGTTCGGGAGCTA	5	234-242	0.705	0.375	0.747
2	Brz0075	6-FAM	GAAGCTGCAAAGGCTGAGT	GGAGGAGAGAGAAGAGCAAGA	8	129-153	0.809	0.727	0.839
	Brz0148	6-FAM	GCTCTTGACCTTGACGATGT	TGCACTTGAGAGAGACGAAA	8	248-274	0.787	0.909	0.800
	Brz0083	HEX	CATGATATTTGCCTGTCAAGG	AGCACCGGTGATGTGAATA	6	233-249	0.765	0.778	0.788
3	Brz0017	HEX	TTCCATTTATTTGCCTGTTCA	ATTTTCCCTATCCGACCTTTC	11	134-160	0.840	1000	0.864
	Brz0116	HEX	TCAAGAAATGGACTCCCAAA	TCTAGGTCATGCAAGCCATT	9	223-271	0.803	0.900	0.827
	Brz0047	6-FAM	TGTGAGACATAAACCATTGGAA	AATGGGTGCTGGAAATGTAAC	7	150-170	0.731	0.556	0.762
4	Brz0021	HEX	CAGCTGAAAGTTCCCAAAAAT	CTGAATGATAAAGGGTGCAAA	9	151-183	0.770	0.400	0.816
	Brz0087	NED	TTCCCCCACTACTCATCTCA	AACAGCACACCGTAGCAAGT	6	239-273	0.716	1000	0.748
5	Brz0065	6-FAM	AGCTAAGCAAATTTCAAGAACG	TAATGTGGAACATTGCCCTAA	12	130-166	0.829	0.700	0.875
	Brz0130	6-FAM	TCCTTTCATGAACCCCTGTA	CATCGCACGCTTATATGACA	9	242-266	0.820	0.636	0.858
	Brz0131	HEX	TGCAATGACATTAAATCAACC	GCTGCAACACAAACAAAATAA	6	254-264	0.712	0.714	0.744
6	Brz0147	HEX	CTGAGGACGCTCCTACTGAA	TTGATTTCAACACCCCAACT	10	240-288	0.825	0.700	0.868
	Brz0031	6-FAM	CCCCCATTTAACACCATAGTT	GCTCAAAATGCAATGTACGTG	7	144-156	0.770	0.667	0.804
7	Brz0177	6-FAM	TGGAGTTGAGGCTTTAGGAA	GTGTTTGGAAACCACTTGCT	6	291-319	0.725	0.125	0.795
	Brz0107	6-FAM	AGAGGAATTGACTTGGAAAAA	GCATGCACGTAAATTTTCACT	6	227-247	0.747	0.444	0.788
	Brz0004	6-FAM	TTGTTGTGGTACACCGGTACT	CAAAACCTGAATCACCATGTC	6	113-155	0.703	0.222	0.745
8	Brz0118	NED	AGGAGGTCCAAATCACCAAT	CGTCAGCAATTCGTACCAC	10	237-263	0.812	0.636	0.849
	Brz0219	HEX	GCAGTTCTTGCTTTTTCAGG	TCTCCTTATGCAAGGCTTC	6	294-304	0.768	0.818	0.778
	Brz0156	6-FAM	GCCATGATGTTTCATTGGTT	TTTTGCACCTTTCATTGCTT	7	239-265	0.752	0.636	0.770
9	Brz0142	6-FAM	GCTGGGTTATGCTAATGCAA	TCAAGCATGAACATTGAAACA	10	241-287	0.823	0.875	0.871
	Brz0180	HEX	CACACGGTCCATCTTGATTT	TCCATAATGCATTGTCTTGAAA	7	285-305	0.751	0.091	0.800
	Brz0089	NED	CAAACCTATTCCACGGTCAA	TGGACAATGCTATTCAAACG	7	224-248	0.710	0.571	0.759
10	Brz0048	HEX	GAATCTAAGCAGCGGATCAAT	TCACAAGAAGGTCCTCACAAG	9	139-161	0.813	0.818	0.839
	Brz0206	NED	GAAGTGGCAAGACACACACA	TGAGCTTTTCGTCTCTCCTG	7	278-302	0.757	0.600	0.783
	Brz0038	6-FAM	CTGAAAATAAGAGCCGTCCAT	ATAAGGTGAGCCACAACTGAG	6	140-154	0.772	0.909	0.778
11	Brz0171	6-FAM	TTGTCTCACTTGTGCACTCC	GCTAGCAGGTAGCAAGATGG	7	312-348	0.725	0.250	0.787
	Brz0015	6-FAM	AATAGAAAACGTGAGCCCATT	TCCACCAATATGATTCAAACG	6	144-156	0.764	0.636	0.783
	Brz0152	NED	ATGCTGCACTTACTGGTTCA	GGCTATCAATTCGAAGACCA	6	228-248	0.748	0.667	0.774

### Transferability to other brachiaria species

A survey on the potential transferability of microsatellite markers generated for ruzigrass to other brachiaria species showed that 90.9% of the 198 polymorphic markers presented amplified PCR products on *Brachiaria brizantha* cv. Marandu, 67.7% on *B. brizantha* cv. Piatã, and 87.9% on *B. brizantha* cv. Xaraés. The percentage of potentially transferable markers to *B. decumbens* cv. Basilisk was 92.9%. Finally, for *Brachiaria humidicola* cv. Tupi, only 42.9% of 198 markers showed amplified PCR products.

## Discussion

A true revolution is taking place on our ability to identify and develop microsatellite markers either for breeding, germplasm characterization, or conservation. The steady decrease in costs for obtaining next-generation sequencing data has made possible for research groups with access to an NGS facility to put a new model of microsatellite development to the test.

Most of the first published papers reporting the use of next-generation sequencing technologies for the development of microsatellite markers used either shotgun pyrosequencing of genomic DNA [8-12], or of enriched libraries [19]. Illumina sequencing was first applied to transcriptome sequencing and assembly, followed by the detection of genic SSR markers [20,21]. Castoe et al. [13] tested the use of Illumina paired-end reads of genomic DNA, without enrichment or assembly of reads, to detect potentially amplifiable microsatellite loci on three different organisms. This approach was also used by O’Bryhim et al. [22] to develop microsatellite markers on an endangered scaleshell species. Castoe’s work does not present any data on the test of synthesized primer pairs. O’Bryhim’s paper reports the test of 48 primer pairs, 16 of which were polymorphic.

We show that reads from an Illumina single-end run, when assembled *de novo* with high levels of stringency, are also suitable for the identification of microsatellite regions. Even though we haven’t tested Castoe’s scripts to detect potentially amplifiable loci from unassembled reads, we believe the assembly process adds a consistent level of sequence quality. That increases the chance of finding good-quality flanking regions for which primer pairs can be designed.

Squirrel et al. [23] used the term “attrition rate” to describe the loss of loci at each step of microsatellite marker development. For traditional projects - which include the construction of clone libraries, the sequencing of clones, microsatellite identification, primer design, and PCR - their estimate based on a review of published papers showed that, on average, 83% of the sequenced clones would be lost due to problems in different steps of the development process.

The application of this criterion to measure how much effort is necessary to develop functional, polymorphic microsatellite markers using genome surveys based on next-generation sequencing depends on the definition of what initial count is used. In our case, depending on the imposed stringency on contig coverage, our initial number of potentially useful, perfect microsatellite markers ranged from 139,098 to 85,567 (at least 10X contig coverage), and finally to 1,135 (100X coverage). If we chose the most stringent parameter, we would expect that from our 1,135 microsatellite-containing sequences, 729 would be suitable for primer synthesis (46% of mean attrition rate on this step), and 365 would be polymorphic (50% mean attrition rate). If we only consider that final step, the expected number of functional polymorphic markers from our set of tested primer pairs would be 135 (starting with our 269 loci). Our observed number of polymorphic markers was higher, 198 of our 269 tested primer pairs were polymorphic (73.6%).

We could apply the attrition rate estimates published by Squirrel et al. to answer one more question: given our final set of functional polymorphic microsatellite loci, how much effort would be necessary in previous steps of marker development if we were using a traditional clone library approach? The answer is that in order to obtain 198 functional polymorphic loci, 1,146 clones from an enriched library would have to be sequenced, 733 microsatellites would have to be identified, and 396 primer pairs would have to be synthesized and tested.

It is obvious that when comparing these estimates, factors such as the abundance of microsatellite regions on the genome of interest are taken for granted. For practical purposes, a more useful comparison would be that between a clone library sequencing method and a next-generation sequencing method on the same organism. In this case, not only the final number of useful markers would be considered, but also costs, time and laboratory workload. Santana et al. [19] have done that for the fungus *Fusarium circinatum*, a pine pathogen. While a single 454 run using pooled ISSR-PCR products detected 231 potentially amplifiable microsatellites (out of 1,692 contigs and singletons), Sanger sequencing of 100 clones containing ISSR-PCR fragments allowed the detection of 8 potentially amplifiable sequences.

We can compare our effort with previous microsatellite development initiatives for other brachiaria species. In *B. brizantha*[15], 96 clones from an enriched library were sequenced, 19 primer pairs were designed and tested, and 13 of those were polymorphic. A new set of 15 polymorphic primers for this species was published by Vigna et al. [16], using the same enriched library. For *B. humidicola*, 384 clones were sequenced, 38 primer pairs were tested, and 27 were polymorphic [17]. A new set of 40 primer pairs was tested by Vigna et al. [18], 38 of which were polymorphic. No microsatellite markers had been developed so far for *Brachiaria ruziziensis*.

It seems, therefore, that the detection and development of microsatellite markers from genome assembled Illumina single-end DNA sequences is highly efficient. This approach should be especially considered for species with limited genomic information.

The need for further germplasm collection expeditions to increase the genetic diversity of *B. ruziziensis* kept in germplasm banks should also be mentioned. It was observed that roughly 30% of the expected allele sizes were not detected on the 11 ruzigrass accessions genotyped in this study. Since the plant used to generate the single-end sequences is derived from a self-pollinated plant collected in the field in Brazil, this data indicates that there is genetic variation in ruzigrass that is out of the allele variation boundaries observed in the analyis of the 11 African-derived genotypes used in this experiment. It is possible that new germplasm collection initiatives in pastures established in the 1960-1970’s in Brazil will identify accessions with useful genetic diversity for ruzigrass breeding programs.

Finally, although we consider the data on transferability of ruzigrass microsatellite markers to other brachiaria species rather preliminary, the higher proportion of successful PCR amplifications on *B. brizantha* and *B. decumbens* cultivars indicates a closer phylogenetic distance between these species and *B. ruziziensis*, when compared with *B. humidicola*.

## Conclusions

We show that the detection and development of microsatellite markers from genome assembled Illumina single-end DNA sequences is highly efficient. The developed markers are readily suitable for genetic analysis and marker assisted selection of *Brachiaria ruziziensis*. The use of this approach for microsatellite marker development is promising for species with limited genomic information, whose breeding programs would benefit from the use of genomic tools. To our knowledge, this is the first set of microsatellite markers developed for this important species.

## Methods

### Sequencing and *de novo* partial assembly of the *B. ruziziensis* genome

*B. ruziziensis* genome sequencing was performed with DNA extracted from a self-pollinated plant (FSS-1 clone), in order to increase homozygosity and, as a consequence, facilitate the *de novo* genome assembly. Sequencing was performed from a genomic DNA fragment library, amplified by cluster generation by bridge PCR, allowing the massive parallel sequencing by synthesis in an Illumina GAII sequencer. Assembly routines were performed on CLC Genomics Workbench software (CLC Bio, Aarhus, Denmark). An assembly mapping was obtained after removing of Illumina adapters and low quality sequences using the CLC trimmer function (default limit = 0.05). The assembly procedure used the parameters Length Fraction (LF) and Sequence Similarity (SIM) between DNA reads, as described by the CLC Genomics Workbench software, with maximum stringency (0.50 LF and 0.80 SIM). The minimum contig length parameter was set to 70 bp.

### Selection criteria for microsatellite loci in *B. ruziziensis*

Microsatellite sequence discovery was carried with Phobos [24]. Initially, we searched for di-, tri-, and tetra-nucleotide loci with perfect repeat motifs on assembled contigs with at least 10X coverage. This allowed a preliminary survey of the most frequent types of repeat motifs on the assembled genome, and the number of repeat motifs for the detected loci. A dataset with contigs >200 bp was then used to map the reads using maximum stringency (100% LF and 100% SIM), in order to minimize the error of consensus sequences while improving the coverage of conserved sequences. With this procedure, the average length of resulting contigs was increased. Perfect microsatellites which occurred in the contigs greater than 200 bp and with coverage above 10x could be recovered using Phobos. A final set of 500 microsatellites with minimum 100x coverage was then selected for analysis and validation (Additional file 1: Table S1). The microsatellite containing sequences received the GeneBank accession numbers KC181352 - KC181851.

In order to test some of these loci on *Brachiaria ruziziensis* germplasm, primer pairs were designed with Primer3Plus [25]. From the initial list of detected microsatellites, we generated a subset of loci which were present on contigs with at least 100X coverage. Two hundred and seventy primer pairs were designed (240 di-nucleotides, 20 tri-nucleotides, and 10 tetra-nucleotides). Fluorescent labels were added to the forward oligos of each primer pair so that multiplexing and genotyping would be performed on an automated DNA sequencer.

### Plant material for SSR genotyping

We tested the synthesized primer pairs on eleven ruzigrass samples - ten accessions from the Embrapa Germplasm Collection and one cultivar (Kennedy). The ruzigrass accessions were selected for this study based on their expected high genetic diversity, since they are progenies of original germplasm accessions collected in the 1980’s in different countries of Africa, where *B. ruziziensis* is endemic [26]. Seeds were germinated and DNA was extracted using a standard CTAB protocol [27] with modifications, as described in [28]. Leaves from five cultivars of other *Brachiaria* species were also collected and had their DNA extracted. These were cultivars Marandu, Piatã and Xaraés (*Brachiaria brizantha*), cultivar Basilisk (*Brachiaria decumbens*) and cultivar Tupi (*Brachiaria humidicola*), all of them registered for commercial cultivation in Brazil. They were genotyped in order to test the transferability of SSR markers designed for *B. ruziziensis* to commercially important polyploid *Brachiaria* species*.* DNA concentrations were measured on a Nanodrop 2000 spectrophotometer (Thermo Scientific, USA), and samples were diluted on TE buffer pH 8.0 to a concentration of 2 ng/μL.

### Genotyping using multiplex panels of SSR markers

Multiplex panels were designed using Multiplex Manager [29]. They included up to three loci per panel, and all loci in each panel had the same microsatellite repeat motif size. PCR’s were carried in a final volume of 5 μL containing 2 ng of genomic DNA, 1X QIAGEN Multiplex PCR Kit Master Mix (QIAGEN), 0.5X Q-Solution (QIAGEN), and 0.2 μM of each primer. Reactions were performed on a Veriti™ Thermal Cycler (Applied Biosystems, USA) using the following amplification program: 95°C for 15 minutes; 30 cycles at 94°C for 30 seconds, 52°C for 90 seconds, and 72°C for 60 seconds; a final extension step at 60°C for 60 minutes. PCR products were diluted with an equal volume of Milli-Q water, added 10 μL of Hi-Di™ Formamide (Applied Biosystems, USA), a ROX-labeled internal size standard, and denatured at 94°C for 5 minutes. Denatured products were injected on an ABI 3730 (Applied Biosystems, USA) automated sequencer. Allele size calling and genotyping were carried with the GeneMapper® Software v4.1 (Applied Biosystems, USA). Automated allelic binning was performed with AlleloBin http://www.icrisat.org/bt-software-d-allelobin.htm, which is based on an algorithm described in [30]. PowerMarker v. 3.25 [31] was used to generate a table of summary statistics for all loci, as well as a database of allelic frequencies.

## Competing interests

The authors declare that they have no competing interests.

## Authors’ contributions

PITS and AMM prepared genomic libraries, worked on genome assembly, detection of microsatellite sequences, primer design, and multiplex panel development. EGG genotyped ruzigrass accessions with microsatellite markers, analyzed genotyping data and performed statistical analyses. MPF helped analyze genotyping data, performed statistical analyses, selected loci for multiplex panels and drafted the manuscript. MEF conceived of and surpervised the study, performed statistical analyses and helped to draft the manuscript. All authors read and approved the final manuscript.

## Supplementary Material

Additional file 1 List of 500 Brz markers, including their primer sequences, melting temperatures, expected product sizes, and repeat motifs. Click here for file

Additional file 2 Descriptive statistics for 198 polymorphic ruzigrass markers, and information on their transferability to other brachiaria species. Click here for file
